# Advanced Estimation of Compressive Strength and Fracture Behavior in Ceramic Honeycombs by Polarimetry Measurements of Similar Epoxy Resin Honeycombs

**DOI:** 10.3390/ma15072361

**Published:** 2022-03-22

**Authors:** David Köllner, Bastien Tolve-Granier, Swantje Simon, Ken-ichi Kakimoto, Tobias Fey

**Affiliations:** 1 Department of Materials Science and Engineering (Glass and Ceramics), Friedrich-Alexander-Universität Erlangen-Nürnberg, Martensstr. 5, D-91058 Erlangen, Germany; david.koellner@fau.de (D.K.); swantje.simon@fau.de (S.S.); 2 Sciences and Technologies Faculty of Limoges, 87060 Limoges, France; bastien.tolve-granier@etu.unilim.fr; 3 Frontier Research Institute for Materials Science, Nagoya Institute of Technology, Gokiso-cho, Showa-ku, Nagoya 466-8555, Japan; kakimoto.kenichi@nitech.ac.jp

**Keywords:** auxetic honeycombs, polarimetry, digital image correlation, ceramic transfer molding, stress prediction

## Abstract

Finding a non-destructive characterization method for cellular ceramics’ compressive strength and fracture behavior has been a challenge for material scientists for years. However, for transparent materials, internal stresses can be determined by the non-destructive photoelastic measurements. We propose a novel approach to correlate the photoelastic stresses of polymer (epoxy resin) prototypes with the mechanical properties of ceramics (alumina). Regular and inverse epoxy honeycombs were 3D-printed via stereolithography with varying structure angles from −35° to 35°, with negative angles forming an auxetic and positive hexagonal lattice. Photoelastic measurements under mechanical loading revealed regions of excess stress, which directly corresponded to the initial fracture points of the ceramic honeycombs. These honeycombs were made by a combination of 3D printing and transfer molding from alumina. The photoelastic stress distribution was much more homogeneous for angles of a smaller magnitude, which led to highly increased compressive strengths of up to 446 ± 156 MPa at 0°. By adapting the geometric structural model from Gibson and Ashby, we showed that we could use a non-destructive technique to determine the compressive strength of alumina honeycombs from the median photoelastic stress measured on similar epoxy honeycomb structures.

## 1. Introduction

Ceramic honeycombs are a unique subgroup of cellular ceramic materials. Due to their specific shape, they exhibit excellent mechanical properties, such as increased flexural strength with a simultaneous low density, and are widely used in aerospace and sandwich constructions [1]. Specific cases are re-entrant honeycombs or auxetic structures, which contract under compression load and expand under tensional load because of a negative Poisson’s ratio [2]. This auxetic behavior is caused by the inward folding of the struts, which results in a material-independent structure effect [3]. A consequence of the auxetic behavior is a higher damage tolerance, higher toughness, and increased sensitivity to sensors [2,4]. In particular, strain amplification can be obtained by uniform lattices (one type of unit cell) [5,6], whereas zero strain perpendicular to the force direction (Poisson’s ratio of zero) can be achieved by combining different unit cells [7]. Meanwhile, the mechanical properties and residual stresses depend on the geometric parameters and loading direction and can be highly anisotropic in-plane and out-of-plane [8,9,10,11]. Of particular interest are the in-plane properties of Poisson’s ratio, Young’s modulus, and strength, which can be modified by geometric parameters as angle, strut thickness, etc. [11,12,13,14]. The mechanical properties (e.g., maximum elongation, compressive strength) of ceramic cellular materials are limited by their low tensile and flexural strength, as the struts in normal and re-entrant honeycombs are loaded in bending and tension [10,15]. Therefore, failure occurs where the maximum tolerable tensile stress of the ceramic induced by the bending moment is exceeded. [15]. Compression and tensile tests combined with digital image correlation (DIC) are typically deployed to track and understand the fracture behavior of complex structures [16,17,18,19]. In contrast to the destructive testing method, non-destructive and non-contact photoelastic measurements can be performed. These are widely used for analyzing internal stresses and fracture processes in glasses and transparent polymers from simple to complex shapes [20,21,22,23,24]. Here, polarized light is shone perpendicularly through the sample, creating a fringe pattern that provides information about local stress states (*σ*) [25,26]. The material-dependent stress optic coefficient *C* is necessary for a quantitative evaluation of the internal stress, which can be determined by measuring the retardation (*δ*) at different forces via Equation (1) [27]. Using the stress-optical coefficient *C*, the fringe order *N*, and the specimen thickness *t*, the material stress fringe value *f_σ_* and the stress can be calculated using Equation (2) [26,28]:

(1) $$∆n=\frac{\delta }{t}=C*\left(\sigma_{11}-\sigma_{22}\right)$$

(2) $$\sigma_{11}-\sigma_{22}=\frac{N*f_{\sigma }}{t}$$

Based on the brittle fracture behavior of the different materials (alumina and epoxy), we present a new approach to determine the mechanical properties of cellular structures based on studies of polymer structures. This is feasible because the structural parameters have a greater impact on the mechanical properties of the honeycomb structures than the material itself.

The advantages of using polymers instead of ceramics are extreme savings in costs, time, and energy. Ceramic components go through various time-consuming processes, such as mass production, molding, post-processing, and high-energy thermal treatment [29]. In contrast, polymer prototypes can be additively and cost-effectively manufactured in a few hours, with no need for extensive post-processing or thermal treatment [30,31]. 

**Figure 1:**
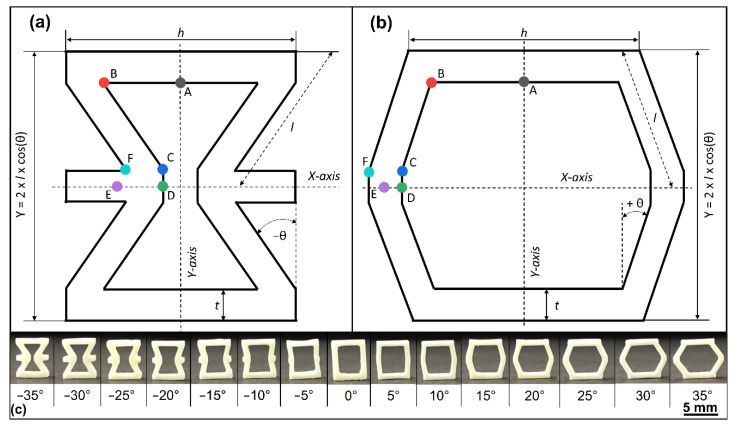
(**a**) Auxetic and (**b**) hexagonal unit cell with symmetry axes (X/Y) and structural parameters: strut thickness *t*, width *h*, angle θ, leg length *l*, and measuring points A−F for photoelastic stress measurement, (**c**) alumina unit cells with varying angle θ between θ = −35° and 35°.

This work shows how photoelastic measurements of polymer prototypes (e.g., honeycombs) can predict the fracture behavior and compressive strength of ceramic honeycombs. For this purpose, hexagonal (θ ≥ 0°) and auxetic (θ < 0°) unit cells (Figure 1) were fabricated from polymers via stereolithography and ceramics via transfer molding. The stress optical coefficient of the epoxy resin was first determined, and then the residual stresses were measured as a function of angle using a strain gauge. For comparison, the compressive strength of the ceramics was determined in a compression test. The fracture behavior was tracked with a high-speed camera and evaluated with an image correlation program. Finally, the results of the photoelastic measurements on the epoxy honeycombs were used to build a fitted model to predict the compressive strength of the ceramic honeycombs.

## 2. Materials and Methods

### 2.1. Sample Preparation

Auxetic and hexagonal unit cells were manufactured by stereolithography and a transfer-molding technique. The geometry, Figure 1a, depended on the width *h*, strut thickness *t*, depth *T*, leg length *l*, and the structure angle θ. In this work, the θ-angle varied between −35 and 35° with a constant height Y of 6.9 mm for the ceramic samples and 31.05 mm for the polymer samples. Ceramic samples have a strut thickness *t* = 0.8 mm, a width *h* = 6.0 mm and a depth *T* = 2.0 mm. Polymer samples have a strut thickness *t* = 5.5 mm, a width *h* = 27.0 mm and a depth *T* = 3.15 mm. The different dimensions of ceramic and polymer samples depend on issues of fabrication and characterization, otherwise fringe patterns would not be resolvable in the polymer samples. For further processing steps, all CAD models were created from their mathematical description using OpenSCAD [32]. 

The polymer samples were printed with an Anycubic Photon Mono (Anycubic, Shenzhen, China) and the resin RF080 (DWS S.r.l., Thiene, Italy) with a z-resolution of 50 µm (x,y resolution = 51 µm). They were cleaned with ethanol and ultrasonic for 5 min and cured for one hour with a UV curing unit (DWS S.r.l., Thiene, Italy). Finally, the samples were polished with a 3 µm diamond suspension (Pureon AG, Lengwil, Switzerland) to achieve a high transparency.

Ceramic samples (Figure 1c) were manufactured by a transfer-molding technique described in detail by Biggemann et al. [33,34]. The positive CAD file was first printed using a stereolithographic 3D printer Digitalwax^®^ 028J, with the resin Fusia DC700 (both: DWS S.r.l., Zanè, Italy) and a z-layer resolution of 20 µm. These were then molded with polydimethylsiloxane (PDMS) (Elastosil M 4643 A/B, Wacker Chemie AG, München, Germany) to obtain the negative silicon casting molds for the transfer-molding process. The alumina feedstock contained 53 vol% alumina CT3000SG (Almatis GmbH, Ludwigshafen, Germany), 40.4 vol% paraffin wax (Granopent P, Carl Roth GmbH, Karlsruhe, Germany) and 4.6 vol% carnauba wax (Naturfarben, Carl Roth GmbH, Karlsruhe, Germany). Transfer molding was performed at 120 °C supported by applying a moderate vacuum (<10 Pa). Debinding (600 °C) and sintering (1600 °C, 2 h) were performed on porous mullite substrates (Annamullit^®^88, Compagnie de Saint-Gobain S.A., Courbevoie, France) with adapted heating and cooling rates between 0.1 and 5 K/min.

### 2.2. Characterization

The photoelastic stress distribution of the polymer samples was measured at a strain of 1.5% (Y-axis) with a Strain Scope Flex (ilis, Erlangen, Germany) at the measuring points A–F (Figure 1). An additional custom control circuit was used to apply a defined force to determine the stress optic coefficient for three rectangular references with the dimensions of 27 mm × 3.15 mm × 3.15 mm. It consisted of a 10 kN load cell (model 88431, Bruster GmbH, Gernsbach, Germany), piezoelectric stack actuator (P-025.80, PI Ceramic GmbH, Lederhose, Germany), power amplifier (PICA HVPZT, PI Ceramic GmbH, Lederhose, Germany), data acquisition system (MGC plus, HBM GmbH, Darmstadt, Germany) and a custom-made LabView program (National Instruments, Austin, TX, USA). With this setup, the load was varied between 0 and 120 N, and the retardation was measured. Using a linear fitting function in Origin 2021 (OriginLab Corporation, Northampton, MA, USA), the gradient was determined, and the stress optical coefficient was calculated via Equation (1). 

The compressive strength was determined using a universal testing machine Instron 5565 (Instron Corp., High Wycombe, UK). Measurements were performed along the Y-axis with a 5 kN and 50 kN load cell and 0.5 mm/min crosshead speed on a minimum of 10 specimens per angle. In addition, the Ximea xiC MC089MG-SY-UB (Ximea GmbH, Münster, Germany) high-speed camera, in combination with a 0,75x TML objective lens (Edmund Optics Inc., Barrington, IL, USA) and the analyzing software VEDDAC 6.4 (Chemnitzer Werkstoffmechanik GmbH, Chemnitz, Germany), was used to track the fracture behavior. 

## 3. Results and Discussion

### 3.1. Influence of the θ-Angle on the Photoelastic Stresses

**Figure 2:**
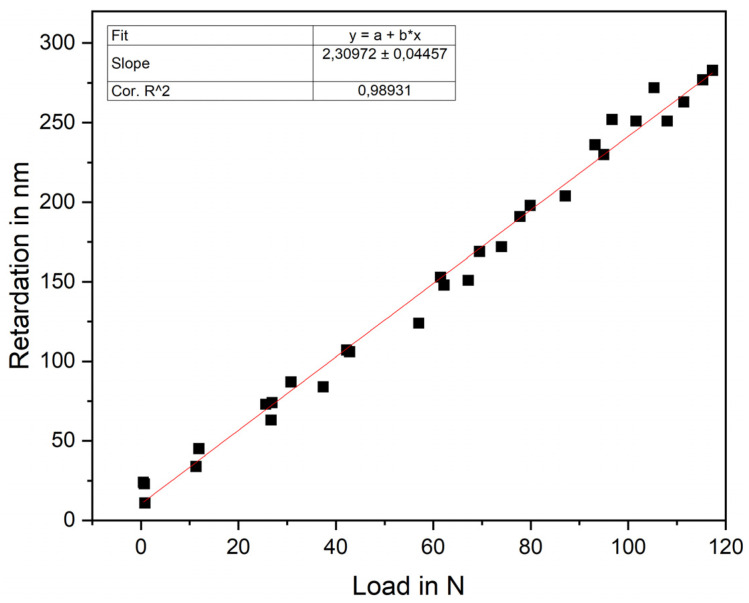
Photoelastic characterization of three rectangular epoxy resin references (27 × 3.15 × 3.15 mm^3^) to measure the retardation dependent on the applied load. Determination of the gradient via a linear fit to calculate the stress-optical coefficient via Equation (1).

**Figure 3:**
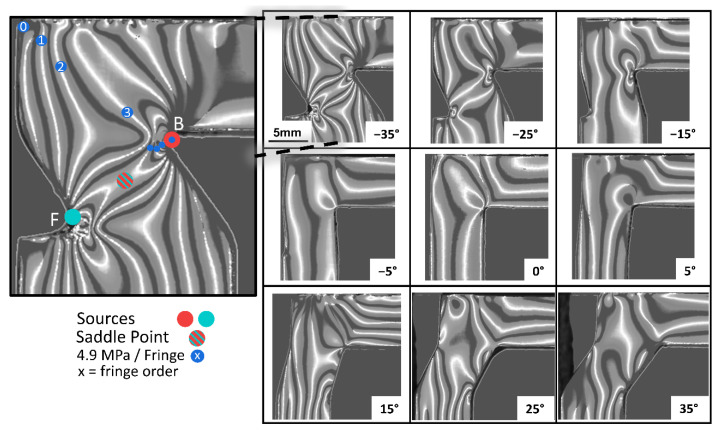
The stress-optical rings (numbered in ascending order) of polymer unit cells are shown for angles θ = −35° and 35°. In the magnification of θ = −35°, the starting points B and F of the rings and the saddle point between both rings are shown. The lowest stress occurred at the saddle point.

To characterize the internal stresses of hexagonal and auxetic unit cells, photoelastic measurements were performed on 3D-printed epoxy polymers. To determine the stress-optical coefficient of epoxy resin, simple rectangular reference specimens were loaded with a force between 0 and 120 N, and the retardation was measured via the strain scope, shown in Figure 2. The stress-optical coefficient of 7.28 TPa^−1^ was obtained via Equation (1) (glass 2–4 TPa^−1^, epoxy resins between 5 and50 TPa^−1^) [26,35,36]. Based on the number of fringes and the stress optical coefficient, the internal stress was determined to be 4.9 MPa per fringe (Equation (2)). To count the fringes, it was necessary to know the sources drawn in the stress-optical images in Figure 3 since the fringes originated from these points, and thus represented the highest order and most-elevated stress. A stress band was formed between the source points with a saddle point in the middle, which has the lowest stress. Perpendicular to the band, the internal stresses decreased again. This band was visible at large negative angles (Figure 3) of −35° and −25° between points B and F, which was the preferred fracture direction. [37,38,39] The stress pattern changed significantly with an increasing angle and showed a linear horizontal and vertical stress alignment, especially between −5° to 5°. With a rising θ-angle over 15°, the overall stress rises again and becomes more inhomogeneous. 

**Figure 4:**
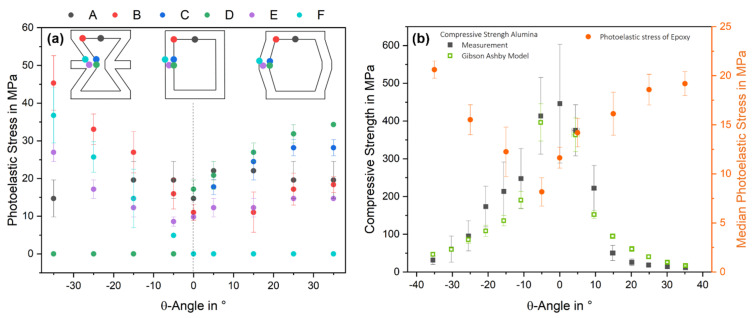
(**a**) Photoelastic stress at defined measurement points for the epoxy resin unit cells depending on the θ-angle (θ ≥ 0 hexagonal, θ < 0° auxetic). (**b**) Compressive strength of alumina ceramic unit cells dependent on the θ-angle and the median photoelastic stresses in the epoxy resin unit cells.

The internal photoelastic stresses at the characteristic points A–F are plotted against θ-angle in Figure 4a. The stress excess point is defined as the maximum stress point per θ-angle, indicating the preferred start position of an initial crack. The maximum stress excess point was determined at point B at an angle of θ = −35° (45.3 ± 7.2 MPa). Upon increasing the θ-angle to −5°, the stress excess point decreased to 19.6 ± 4.9 MPa by simultaneously changing from point B to A. Starting from 0°, the structure changed from auxetic to hexagonal, causing a transition of the stress excess point from A to D, beginning with the minimum stress excess at 0° (17.2 ± 2.5 MPa). Subsequently, the stress excess point increased to 35° at 34.3 ± 2.1 MPa. Consequently, it was possible to adjust the position of the stress excess point and the value over a range of 28 MPa by controlling the θ-angle, which led to a modification of the fracture behavior. 

The median photoelastic stress was also determined as a function of θ and is shown in Figure 4b. From the maximum of 20.6 ± 0.9 MPa at −35°, the median photoelastic stress linearly decreased to 8.2 ± 1.5 MPa at −5°. Subsequently, the mean photoelastic stress hyperbolically increased to 19.2 ± 1.2 MPa at 35°. This confirmed the optical results from Figure 3 that the absolute stress was reduced, and the stress distribution was more homogeneous with smaller absolute values of the angle. Due to both observations, it could be predicted that the compressive strength should be significantly increased at low θ-angles (−5° to 5°).

### 3.2. Structural Influence on Mechanical Properties

**Figure 5:**
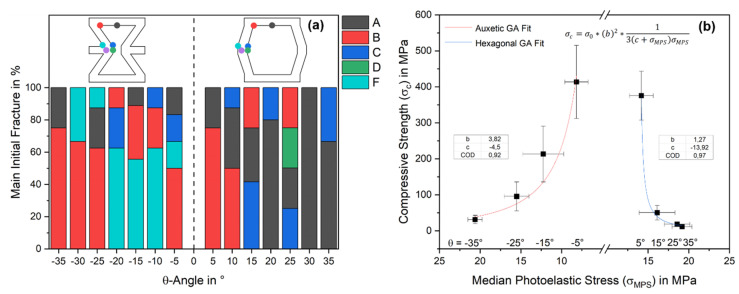
(**a**) Main initial fracture in ascending probability of the alumina samples characterized by DIC (**b**). Compressive strength of ceramic unit cells dependent on the median photoelastic stress of the epoxy unit cells and fitted correlation by an adapted Gibson and Ashby (GA) model: Equations (3) and (4).

The fracture behavior postulated from the photoelastic measurements was evaluated on ceramic specimens. The compressive strength as a function of the θ-angle is shown in Figure 5b. As expected, the compressive strength increased with a smaller θ-angle from 31.57 ± 11.73 MPa at −35° to 446.07 ± 156.94 MPa at 0°, equal to an increase of factor 14. The compressive strength dropped again up to 35° and 11.57 ± 3.47 MPa, representing the lowest value. Overall, this trend followed the theoretical strength, calculated using Equation (3) of Gibson and Ashby et al. [15], which depends on the structural parameters *t, l, h*, θ, and the compressive strength of the material *σ_0_*, which is 2650 MPa for alumina [29]. Here, the exact geometric values of each sample were used to generate a standard deviation of the theoretical calculation. At an angle of 0°, no strength can be calculated, as the function tends to infinity at θ = 0°:

(3) $$\sigma_{c}=\sigma_{0}*{\left(\frac{t}{l}\right)}^{2}*\frac{1}{3\left(\frac{h}{l}+\sin\theta \right)\sin\theta }$$

The compressive strength of the auxetic unit cells (θ < 0°) is higher than the hexagonal unit cells (θ ≥ 0°). This occurs in the model as well as in the experimental results. This can be attributed to the deformation behavior, which can be bending, stretching and hinging, where hinging is the bending of the struts at the vertices, leading to a θ-angle change. If the material is stiff and has a high Young’s modulus, such as alumina, hinging is the dominant deformation mechanism. Depending on the angle, the hinging at the stress excess points B and F generate compressive stresses for θ < 0° (auxetic) and tensile stresses for θ > 0° (hexagonal) [10,12,40,41]. As is generally known, the ratio between tensile, flexural, and compressive strength is 1:2:10, which allows ceramics to withstand higher compressive stresses than tensile stresses [29]. The opposite stress state in the critical points, coupled with the significantly higher tolerable compressive stress, results in higher strengths of the auxetic honeycombs compared to the hexagonal honeycombs.

The fracture behavior was recorded during the compression test and evaluated using DIC, determining the initial fracture point for five samples at each angle. Figure 5b shows the main initial fracture in ascending probability. The auxetic unit cells (θ < 0°) broke most frequently at points B and F. The hexagonal unit cells also show a preferred fracture at point B up to θ = 10°. For θ > 10°, the fracture behavior changes, and the break most frequently starts at points A and C, with clearly different probabilities. Consequently, the hexagonal structures show a more diverse fracture pattern than the auxetic.

Comparing these results with the photoelastic measurements of the epoxy resin, the highest photoelastic stresses in the epoxy correlate with the initial breakpoints of the alumina. In the auxetic range, the photoelastic measurements determined B and F as the primary initial breakpoints for the alumina, which was confirmed by the compression test. In contrast, the fracture in the hexagonal region (θ > 0) should primarily start at points D and C. However, this only takes place at 15° and 25°, with a lower probability of under 50%. Especially at 20°, 30°, and 35°, there is no direct correlation between fracture patterns and photoelastic stress. In these cases, the primary fracture starts at point A but shows only half of the photoelastic stress values compared to points C and D. As mentioned before, the stress state could influence the fracture behavior. Points C and D are loaded primarily in bending and shear, while point A is loaded only in tension [10], which would explain the preferential fracture at Point A. 

Overall, there is an excellent agreement between the initial fracture origins of the ceramic and the highest photoelastic stresses of the epoxy, especially in the auxetic range. With the additional knowledge of discrete stress states, it is also possible to precisely predict the crack initiation location in the entire angular spectrum. Since alumina and epoxy are brittle and exhibit the same fracture behavior, the epoxy’s photoelastic measurement is additionally correlated with the alumina’s compressive strength.

The correlation between the epoxy’s median photoelastic stress (*σ_MPS_*) and the alumina’s compressive strength (*σ_c_*) is shown in Figure 5b and can be described by Equation (4). This is an adaptation of the Gibson and Ashby model (Equation (3)). The median photoelastic stress replaces the angle θ, and the structural parameters are replaced by the empirical constants b and c. The fitted constants in the auxetic range are b = 3.82 MPa and c = −4.5 MPa by R^2^ = 0.92 and for the hexagonal b = 1.27 MPa and c = −13.92 MPa by R^2^ = 0.97. In both cases, the model is suitable for predicting the strength of ceramics from photoelastic measurements of epoxy resin. This also demonstrates that the properties are purely influenced by the structure, and they are independent of the materials if they show the same fracture and stress behaviors.

(4) $$\sigma_{c}=\sigma_{0}*{\left(b\right)}^{2}*\frac{1}{3\left(c+\sigma_{MPS}\right)\sigma_{MPS}}$$

(5) $$b_{auxetic}=3*b_{hexagon}$$

(6) $$c_{auxetic}=\frac{1}{3}*\;c_{hexagon}$$

Moreover, the empirical constants b and c can be converted from the hexagonal model to the auxetic model and vice versa by Equations (5) and (6). As a result, it is necessary to measure only one honeycomb type (auxetic or hexagonal) to modify the model for other material systems, simultaneously reducing the workload by half. 

In general, two essential predictions for the mechanical properties of ceramic components can be made from the photoelastic measurements of polymer prototypes. Firstly, by adapting the model of Gibson and Ashby, it is possible to accurately predict and calculate the compressive strength dependent on the photoelastic stress for auxetic and hexagonal ceramic honeycombs. Secondly, preferred fracture points can be localized by identifying points of excessive stress by photoelastic measurements. This can also be used to define and control the fracture behavior and direction in ceramic honeycombs by mixing different unit cells, since crack propagation would run along the stress excess points. In summary, the photoelastic measurement of polymer prototypes provides a new non-destructive technique to indirectly investigate the compressive strength and fracture behavior of ceramic honeycombs without producing high quantities of ceramic samples.

## 4. Conclusions

This work provided a novel non-destructive indirect method of calculating the compressive strength of ceramic honeycombs from photoelastic stresses by measuring only similar polymer honeycombs.

- Hexagonal and auxetic honeycomb unit cells were fabricated from epoxy and alumina with an θ-angle of −35 to +35;
- Critical stress points were identified by photoelastic measurements of epoxy, which closely matched those fracture points in the alumina determined by DIC;
- Smaller absolute angles showed more homogeneous stress distributions, which were also reflected in the compressive strengths of the alumina, with a maximum of 446 ± 156 MPa at 0°
- The most important achievement was the correlation of the photoelastic measurement of the epoxy with the compressive strength of the alumina by adapting the model from Gibson and Ashby.

## Data Availability

Data sharing is not applicable to this article.
